# Bone bruise distribution predicts anterior cruciate ligament tear location in non‐contact injuries

**DOI:** 10.1002/jeo2.12034

**Published:** 2024-05-13

**Authors:** Steffen T. Ubl, Romed P. Vieider, Jesse Seilern und Aspang, Christian Gaebler, Hannes Platzgummer

**Affiliations:** ^1^ Department of Orthopaedic Surgery, Trauma Surgery and Sports Medicine, Cologne Merheim Medical Center Witten/Herdecke University Cologne Germany; ^2^ Department of Sports Orthopaedics, Klinikum Rechts der Isar Technical University of Munich Munich Germany; ^3^ Department of Orthopaedic Surgery, Grady Memorial Hospital Emory University School of Medicine Atlanta Georgia USA; ^4^ Sportambulatorium Wien – Zentrum für Orthopaedie und Sportchirurgie (ZOS) Vienna Austria; ^5^ Division of Neuroradiology and Musculoskeletal Radiology, Department of Biomedical Imaging and Image‐guided Therapy Medical University of Vienna Vienna Austria

**Keywords:** ACL injury, biomechanic, bone contusion, bone oedema, knee, risk factor

## Abstract

**Purpose:**

It is unclear whether different injury mechanisms lead to divergent anterior cruciate ligament (ACL) tear locations. This study aims to analyse the relationship between bone bruise (BB) distribution or depth and ACL tear location.

**Methods:**

A retrospective analysis of 446 consecutive patients with acute non‐contact ACL injury was performed. Only patients with complete ACL tears verified during subsequent arthroscopy were included. Magnetic resonance imaging (MRI) was used to classify BB location, BB depth, ACL tear location and concomitant injuries (medial/lateral meniscus and medial/lateral collateral ligament). Demographic characteristics included age, gender, body mass index (BMI), type of sport and time between injury and MRI. Multiple linear regression analysis was used to identify independent predictors of ACL tear location.

**Results:**

One hundred and fifty‐eight skeletally mature patients met the inclusion criteria. The presence of BB in the lateral tibial plateau was associated with a more distal ACL tear location (*β* = −0.27, *p* < 0.001). Less BB depth in the lateral femoral condyle showed a tendency towards more proximal ACL tears (*β* = −0.14; *p* = 0.054). Older age predicted a more proximal ACL tear location (*β* = 0.31, *p* < 0.001). No significant relationship was found between ACL tear location and gender, BMI, type of sport, concomitant injuries and time between injury and MRI.

**Conclusion:**

ACL tear location after an acute non‐contact injury is associated with distinct patterns of BB distribution, particularly involving the lateral compartment, indicating that different injury mechanisms may lead to different ACL tear locations.

**Level of Evidence:**

Level III.

**Abbreviations**
ACL: anterior cruciate ligament
BB: bone bruise
BMI: body mass index
ICC: intraclass correlation coefficient
LCL: lateral collateral ligament
LFC: lateral femoral condyle
LTP: lateral tibial plateau
MCL: medial collateral ligament
MFC: medial femoral condyle
MRI: magnetic resonance imaging
MTP: medial tibial plateau
WORMS: whole‐organ MRI score

## INTRODUCTION

Due to the high incidence of non‐contact anterior cruciate ligament (ACL) injuries, there exists major interest in describing the injury mechanism [7, 11, 33, 34], as well as intrinsic risk factors such as morphological, hormonal and neuromuscular factors and extrinsic risk factors such as playing surface, shoe–surface interaction and meteorological conditions [3, 4, 11, 27, 31, 34]. While knowledge of the injury mechanism of non‐contact ACL ruptures is increasing, little is known about its association with different ACL tear locations. Meanwhile, interest in ACL tear location has continued to grow as it is a major indication for successful primary ACL repair [18]. Sherman et al. defined a classification system for various tear locations of the ACL describing four tear types in the proximal half of the ACL [36]. This system has been modified to a clinically applicable grading system [20]. While current biomechanical studies investigating the correlation between loading mechanism and tear location have yielded inconclusive results due to the limited reproducibility of in vivo tear locations [17], it has been hypothesized that the tear location may depend on the severity or the exact biomechanical loading pattern of the knee joint [20, 41]. It is challenging to achieve a complete representation of the injury mechanism that considers all influencing factors such as external loading, muscle forces and neuromuscular control, joint geometry, ACL loading patterns and other risk factors in a single research approach. Therefore, it is important to examine and combine all given aspects [15]. It is well known that bone bruises (BBs) frequently occur after an acute ACL injury and may provide insights into the injury mechanism [29, 45]. Seen on magnetic resonance imaging (MRI), BB is considered as a footprint of the impact [32, 35] and gives information about the position of the knee near the time of ACL tear [13, 28]. Furthermore, the prevalence and size of BB are thought to be associated with the energy level of the injury [43]. Thus, several BB patterns have been described and related to the injury mechanism and severity of the knee joint [10, 12, 37, 38].

The aim of this study was to assess whether ACL tear location in acute non‐contact ACL injuries can be elucidated by distinct BB patterns in skeletally mature patients. The authors hypothesized that there would be differences in BB location and depth in relation to ACL tear location after acute non‐contact ACL tear, controlling for age, gender, body mass index (BMI), type of sport, concomitant injuries of the medial or lateral meniscus and the medial (MCL) or lateral collateral ligament (LCL) and time between injury and MRI.

## MATERIALS AND METHODS

### Study population

After institutional review board approval (2354/2020), all consecutive patients who underwent ACL surgery by a single surgeon (CG) at our institution between 1 December 2016 and 30 November 2020 were retrospectively identified via medical record abstraction. Table 1 shows the justified exclusion criteria. Non‐contact injuries were defined as the absence of physical contact with another person or object at the time of injury [1, 15]. Chronic tears were defined as a failed non‐operative therapy prior to surgical treatment. Partial ACL tears were identified during arthroscopy.

**Table 1:** Summary of the exclusion criteria and associated reasons.

Exclusion criteria	Reason
Contact injury	Deviating BB distribution [25]
Partial ACL tears	Deviating BB distribution [44]
Open growth plates	Deviating BB distribution [26]
Previous ipsilateral knee joint surgery	Possible deviating BB distribution
Chronic ACL tears	Missing or changed BB depth
Missing preoperative MRI	Inadequate or non‐determinable ACL tear location
Insufficient MRI quality (less than 1.5 T)	
ACL tear location could not be determined	
Missing T2 or proton density fat‐suppressed sequences in sagittal and coronal planes	Inadequate or non‐determinable BB location
Slice thickness >4 mm	
Insufficient MRI quality (less than 1.5 T)	
Interval between injury and MRI >4 weeks	BB depth changes [39]

Abbreviations: ACL, anterior cruciate ligament; BB, bone bruise; MRI, magnetic resonance imaging; T, Tesla.

### Data collection and MRI analysis

**Figure 1:**
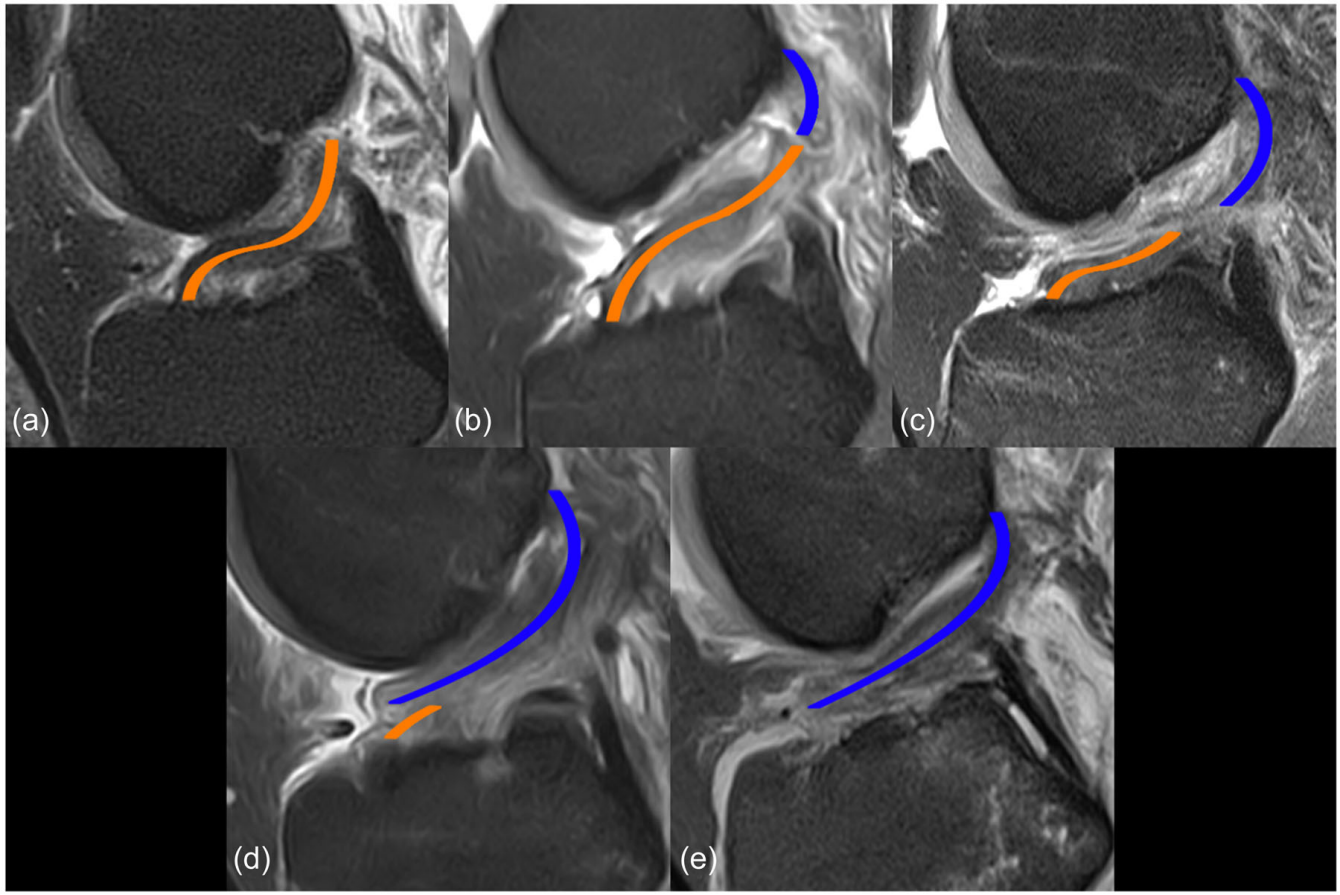
Illustration of (a) type I, (b) type II, (c) type III, (d) type IV, and (e) type V anterior cruciate ligament (ACL) tears using the measurement technique of Vermeijden et al. [40]. The distal remnant (orange lines) was determined by measuring the distance from the anterior tibial insertion of the ACL to the centre of the proximal end of the distal remnant. The length of the proximal remnant (blue lines) was measured from the most proximal point of the femoral insertion site to the centre of the distal end of the proximal remnant. In some cases, depending on the course of the remnants, the measurements were performed in several slices.

Demographic characteristics included age, gender, BMI, type of sport and time between injury and MRI. ACL tear type was graded into five different tear types according to the modified Sherman classification [20]. The ACL tear location measurement technique of Vermeijden et al. (Figure 1) was used to achieve higher inter‐rater reliability [40]. ACL tear location was classified by dividing the distal remnant length by the total (proximal and distal) remnant length. Type I tears were classified as proximal avulsions with >90% distal remnant length, type II tears were classified as proximal tears with a distal remnant length of 75%–90%, type III tears were classified as midsubstance tears with a distal remnant length of 25%–75%, type IV tears were classified as distal tears with a distal remnant length of 10%–25% and type V tears were classified as distal avulsions with <10% distal remnant length.

All available sequences and planes were considered for BB analysis, especially fat‐suppressed, T2‐weighted or proton density‐weighted sequences. BB was defined as a localized and nonlinear area of increased signal intensity on T2‐weighted or decreased signal intensity on T1‐ or proton‐density‐weighted sequences involving the subcortical bone beneath the tibiofemoral articular surface [23]. The presence of BB was documented separately for the following four anatomical sites: lateral femoral condyle (LFC), medial femoral condyle (MFC), lateral tibial plateau (LTP) and medial tibial plateau (MTP). Each anatomical site was divided into an anterior, central and posterior segment using a modified technique [5] of the whole‐organ MRI score (WORMS) [30]. For the final analysis, only the presence and depth of BB in the aforementioned four anatomic sites were considered, as BB was mostly found in the same segments. The BB prevalence and depth within the segments are shown in Supplementary File 1.

**Figure 2:**
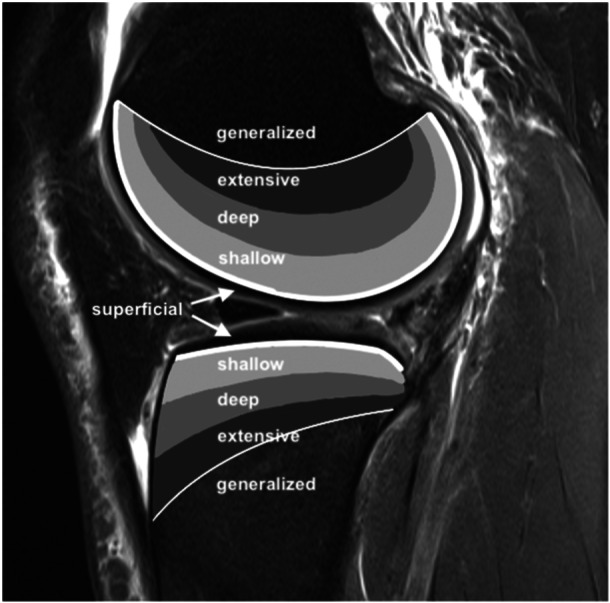
Classification of bone bruise depth according to Brittberg and Winalski [8] categorized as superficial (beneath the subchondral bone), shallow (up to one third of the distance to the physeal scar), deep (between one third and two thirds of the distance to the physeal scar), extensive (at least two thirds of the distance to the physeal scar, but not beyond) or generalized (beyond the physeal scar).

The BB depth was graded separately for each anatomical site and segment according to the International Cartilage Repair Society knee cartilage lesion mapping system [8] (Figure 2).

MCL and LCL injuries were classified into three grades (I–III) according to Mirowitz and Shu [24]. Only grade II and grade III injuries were considered as relevant structural damage and therefore included in further analysis. Additionally, only grade III lesions of the medial and lateral meniscus according to Lotysch et al. [21] were included. Image analysis was performed using only true sagittal, axial and coronal slices.

### Measurement protocol

MRI scans were imported into the DICOM medical image viewer HOROS (Version 4.0.0, Nimble Co LLC d/b/a, Purview, Annapolis, USA) and reviewed by three raters, all blinded to demographic data: a board‐certified radiologist with 13 years of experience in musculoskeletal radiology (HP), a board‐certified trauma surgeon (CG) with 29 years of experience in orthopaedic and trauma surgery and 20 years of experience in ACL preservation techniques and a medical research fellow (STU). Each parameter was assessed in separate sessions. The results of the board‐certified radiologist (HP) were used for the statistical analysis. For the inter‐rater reliability analysis, 30 randomly selected patients were compared.

### Statistical analysis

Data were analysed using SPSS 29.0.1.0 (IBM‐SPSS). Descriptive statistics were performed for each group (tear type). Inferential statistics were performed on three groups (ACL tear type I–III) and outliers were excluded using boxplots (ACL tear types IV and V). All continuous variables underwent Shapiro–Wilk tests to assess distribution; due to non‐normal distribution (*p* ≤ 0.05), non‐parametric tests were used. The Pearson *χ* ^2^ test or the Fisher exact test was used to compare nominal variables between ACL tear type groups, and the Kruskal–Wallis test with post hoc Dunn's test was used for continuous variables. The univariate analysis included the Mann–Whitney *U* test for nominal and continuous variables. The Pearson correlation coefficient (r) was determined between continuous variables due to a sample size of *n* > 30 and the Kendall rank correlation coefficient (*r*) between continuous and ordinal variables. Multivariate linear regression analysis with bootstrapping (1000 samples) was performed to identify independent predictors of the criterion ACL tear location. A post hoc power analysis was performed using G*Power (Version 3.1.9.7, Kiel, Germany). High statistical power (1 − *β* =0 .99) was achieved with a coefficient of determination of *R* ^2^ = 0.23, a sample size of *n* = 155 and a significance level of *α* = 0.05 with four predictors. Inter‐observer agreement was tested using Fleiss kappa (*κ*) for nominal and ordinal data and intraclass correlation coefficient (ICC) for metric data. Observer agreement using Kappa statistics was defined as almost perfect (*κ* > 0.80), substantial (*κ* = 0.80–0.61), moderate (*κ* = 0.60 0.41), fair (*κ* = 0.40–0.21) or poor (*κ* < 0.21) [16]. In addition, ICC has been defined as poor (<0.5), moderate (0.5–0.75), good (0.75–0.9) and excellent (>0.9) [14].

## RESULTS

### Demographic characteristics

**Figure 3:**
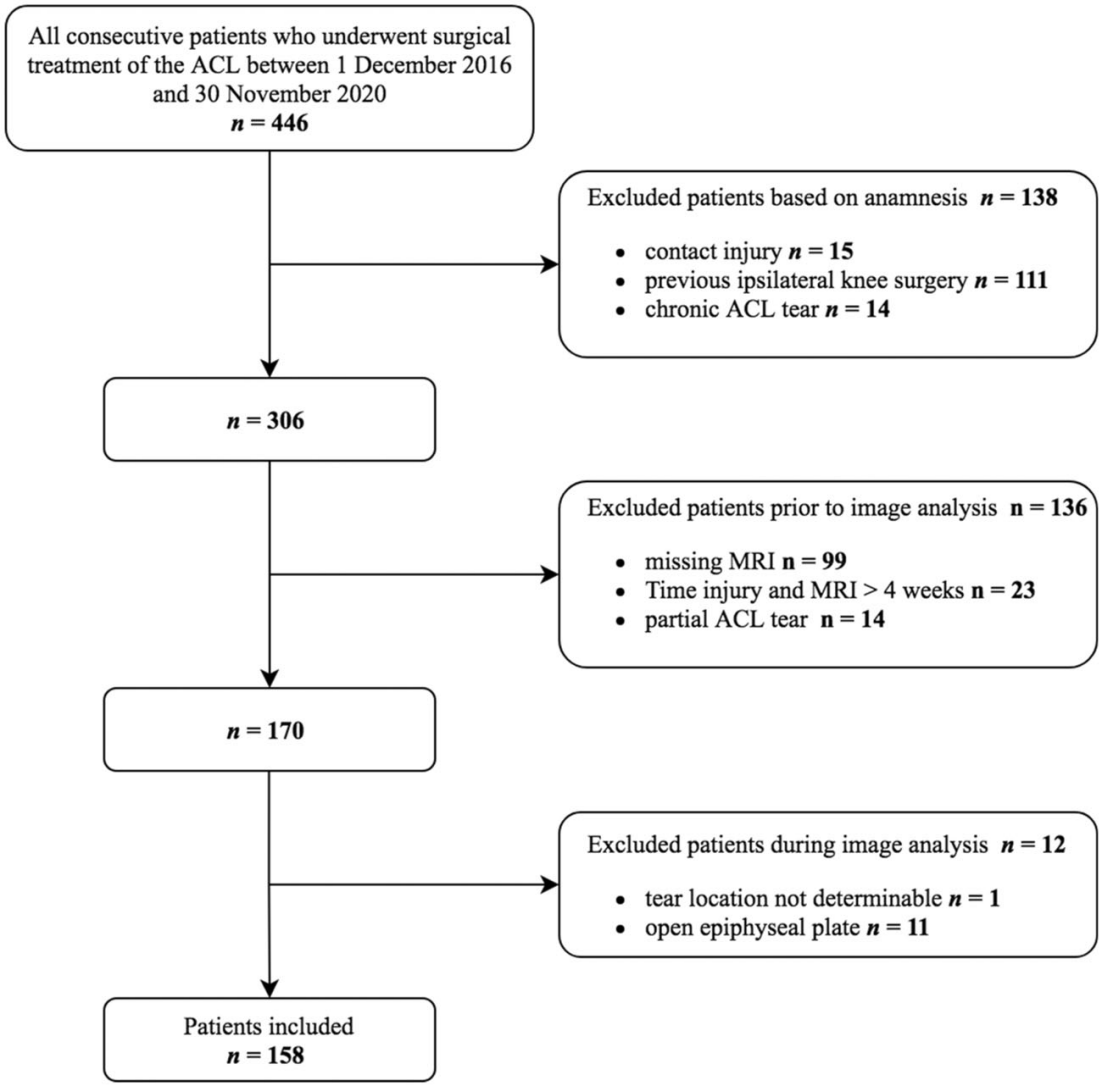
Flowchart of included and excluded patients. ACL, anterior cruciate ligament; MRI, magnetic resonance imaging.

A total of 158 patients were available for final analysis after application of exclusion criteria (Figure 3). Details on patient demographics are presented in Table 2.

**Table 2:** Demographic data of all patients at the time of injury.

	Total (*n* = 159)	ACL tear type					*p* Value
		Type I (*n* = 11)	Type II (*n* = 28)	Type III (*n* = 116)	Type IV (*n* = 1)	Type V (*n* = 2)	
Age (years)	32.0 [14–61]	43.0 [16–56]	44.5 [15–61]	31.0 [14–55]	28.0	17.5 [16–19]	0.001**
BMI (kg/m^2^)	24.0 [17.3–42.5]	22.3 [20.5–24.9	24.0 [18.1–34.1]	24.2 [17.3–42.5]	22.1	25.8 [21.7–29.9]	n.s.
Time injury to MRI (days)	3.0 [0–28]	3.0 [0–10]	3.5 [0–10]	3.0 [0–28]	10	2.5 [1–4]	n.s.
Gender							
Female	82 (51.9)	5 (45.5)	14 (50.0)	63 (54.3)	0	0	n.s.
Male	76 (48.1)	6 (54.5)	14 (50.0)	53 (45.7)	1 (100)	2 (100)	
Type of sport							
Skiing	65 (41.1)	5 (45.5)	15 (53.6)	44 (37.9)	0	1 (50.0)	
Soccer	26 (16.5)	3 (27.3)	4 (14.3)	17 (14.7)	1 (100)	1 (50.0)	
Football	8 (5.1)	0	0	8 (6.9)	0	0	
Handball	6 (3.8)	0	0	4 (3.4)	0	0	n.s.
Basketball	5 (3.2)	0	0	6 (5.2)	0	0	
Tennis	5 (3.2)	0	2 (7.1)	3 (2.6)	0	0	
Gymnastics	4 (2.5)	1 (9.1)	0	4 (3.4)	0	0	
others	39 (24.7)	2 (18.2)	7 (25.0)	30 (25.9)	0	0	
Medial meniscus injury	58 (36.7)	6 (54.5)	9 (32.1)	42 (36.2)	0	1 (50.0)	n.s.
Lateral meniscus injury	26 (16.5)	1 (9.1)	5 (17.9)	19 (16.4)	0	1 (50.0)	n.s.
MCL injury	21 (13.3)	2 (18.2)	5 (1.9)	13 (11.2)	0	1 (50.0)	n.s.
LCL injury	5 (3.2)	0	1 (3.6)	4 (3.5)	0	1 (50.0)	n.s.

*Note*: Statistic comparison between ACL tear types I–III after exclusion of outliers (ACL tear types IV and V). Values given as median [range] for continuous variables and n (%) for categorical variables.

Abbreviations: ACL, anterior cruciate ligament; LCL, lateral collateral ligament; MCL, medial collateral ligament.

** *p* ≤ 0.01.

### ACL tear location

The median ACL tear location was 68.2% distal remnant length (range 0%–100%). Overall, 7.0% of the patients had a type I tear, 17.7% had a type II tear, 73.4% had a type III tear, 0.6% had a type IV tear and 1.3% had a type V tear of the ACL. When comparing the different ACL tear types, there was a significant difference (*p* = 0.001) in age at the time of injury (Table 2). Patients with a type III tear were significantly older than those with a type II tear (*p* = 0.005).

### Bone bruise distribution and depth

**Figure 4:**
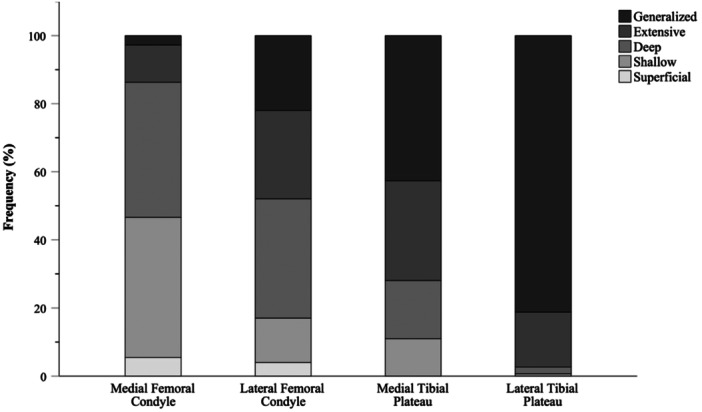
Frequency of bone bruise depth in analysed anatomical sites.

Overall, BB was found in 96.2% of patients. The most commonly affected anatomical site was the LTP (94.3%), followed by the LFC (63.3%), the MTP (51.9%) and finally the MFC (46.2%). Figure 4 shows that BB depth is greater in LFC and LTP than in MFC and MTP.

### Observer agreement

Inter‐rater reliability for ACL tear location was excellent (ICC = 0.92). Inter‐rater agreement regarding BB presence at each anatomical site was graded as almost perfect (*κ* = 0‐95–1.00) and for BB depth at each anatomical site as substantial (*κ* = 0.68–0.75). For concomitant injuries, the agreement was graded as substantial for LCL (*κ* = 0.64) and lateral meniscus (*κ* = 0.71) and as almost perfect for MCL (*κ* = 0.83) and medial meniscus (*κ* = 0.94).

### Univariate analysis

Absence of BB in the LTP was associated with a significantly more proximal ACL tear location (*p* < 0.001). In addition, greater BB depth in the LFC (*r* = −0.27, *p* ≤ 0.001) and MTP (*r* = −0.19, *p* = 0.029) was associated with a significantly more distal ACL tear location. Correlation analyses showed a positive correlation between more proximal ACL tear location and increasing age at the time of injury (*r* = 0.39, *p* ≤ 0.001). No significant relationship was found between ACL tear location and BB depth in MFC or LTP, BMI and time between injury and MRI (*p* ≥ 0.05). Furthermore, the presence of BB in the LFC, MFC and MTP, gender, type of sport and concomitant injuries did not show any significant differences in ACL tear location (Table 3).

**Table 3:** Results of univariate analyses of potential predictors of ACL tear location.

	*n* (%)	ACL tear location, %	*p* Value
		Median [range]	
Gender			
Female	82 (52.9)	69.4 [36.0–100]	n.s.
Male	73 (47.1)	64.6 [40.2–100]	
Medial meniscus injury			
Yes	57 (36.8)	68.6 [36.0–100]	n.s.
No	98 (63.2)	67.7 [40.2–100]	
Lateral meniscus injury			
Yes	25 (16.1)	68.6 [42.7–93.1]	n.s.
No	130 (83.9)	68.3 [36.0–100]	
MCL injury			
Yes	20 (12.9)	67.4 [40.2–100]	n.s.
No	135 (81.1)	68.3 [36.0–100]	
LCL injury			
Yes	5 (3.2)	69.5 [58.7–86.8]⁠	n.s.
No	150 (96.8)	68.3 [59.9–75.1]	
Bone bruise MFC			
Yes	71 (45.8)	67.9 [41.3–100]	n.s.
No	84 (54.2)	69.2 [36.0–100]	
Bone bruise MTP			
Yes	79 (51.0)	65.2 [42.5–100]	n.s.
No	76 (49.0)	69.2 [36.0–100]	
Bone bruise LFC			
Yes	98 (63.2)	65.8 [36.0–100]	n.s.
No	57 (36.8)	70.1 [40.2–100]	
Bone bruise LTP			
Yes	146 (94.2)	67.1 [36.0–100]	<0.001**
No	9 (5.8)	87.3 [70.4–100]	

Abbreviations: ACL, anterior cruciate ligament; LCL, lateral collateral ligament; LFC, lateral femoral condyle; LTP, lateral tibial plateau; MCL, medial collateral ligament; MFC, medial femoral condyle; MTP, medial tibial plateau.

** *p* ≤ 0.01.

### Multivariate analysis

Multivariate linear regression analysis (*R* ^2^ = 0.23, *F* = 12.67, *p* < 0.001) with bootstrapping (1000 samples) was performed for four potential predictors that had *p* values <0.05 in the univariate analysis. In the multivariate analysis (Table 4), two predictors showed a significant influence on the ACL tear location (*p* ≤ 0.001). The presence of BB in the LTP predicted a more distal ACL tear location (*β* = −0.27, *p *< 0.001). Furthermore, there was a tendency towards a more distal ACL tear location with greater BB depth in the LFC (*β* = −0.14, *p* = 0.054). Older age at the time of injury was associated with a more proximal ACL tear location (*β* = 0.31, *p* < 0.001).

**Table 4:** Coefficients of the studied predictors in relation to the criterion (ACL tear location) of the multiple regression analysis with bootstrapping (1000 samples).

Predictors	Regression coefficient *B* [95% CI]	*β*	*p* Value
Age at time of injury (continuous)	0.33 [0.017–0.49]	0.31	<0.001**
Presence of BB in LTP (no vs. yes)	−15.13 [−25.05 to −4.91]	−0.27	<0.001**
BB depth in MTP (≤extensive vs. generalized)	−5.29 [−6.49 to 2.38]	−0.07	0.340
BB depth in LFC (≤extensive vs. generalized)	−2.24 [−10.55 to 0.07]	−0.14	0.054*

Abbreviations: ACL, anterior cruciate ligament; B, unstandardized β coefficient; β, standardized β coefficient; BB, bone bruise; CI, confidence interval; LFC, lateral femoral condyle; LTP, lateral tibial plateau; MTP; medial tibial plateau.

** *p* ≤ 0.01

* *p* ≤ 0.10 (tendency).

## DISCUSSION

The main findings of this study were that the absence of BB in the LTP was associated with a more proximal ACL tear location. In addition, there was a tendency towards a more proximal ACL tear location with less BB depth in the LFC. Furthermore, this study supports recent data [40, 41] that older age at the time of injury is associated with a more proximal ACL tear location and that there is no relationship between the ACL tear location and gender, BMI, type of sport, concomitant injuries of the menisci, MCL and LCL and the time between injury and MRI not exceeding 4 weeks.

A recent study showed that older age is associated with a more proximal ACL tear type; type I tears were more common in patients over 35 years of age, whereas type III and type V tears were more common in patients under 35 years of age [20]. Vermeijden et al. confirmed this relationship using remnant length measurements [40]. The present study verifies the relationship between age and ACL tear location. Possible reasons for this could be a decrease in ACL vascularity and an associated mucoid degeneration or a difference in the injury mechanism [40, 41].

In support of the latter hypothesis, this study showed that the presence of BB in the LTP was associated with a more distal ACL tear location. In addition, there was a tendency towards a more proximal ACL tear location with less BB depth in the LFC.

The presence of BB in the lateral compartment is related to increased knee valgus loading [10, 35, 45], and BB depth is considered to be an indicator of the energy at the time of the injury [2, 6, 42, 43]. Thus, it is possible that proximal ACL tears are the result of low‐energy trauma or a lower knee valgus angle, resulting in less compression force between the LFC and the LTP. This is consistent with the theory of Van der List et al. that the ACL tends to avulse from the femoral origin in low‐energy injuries, whereas in high‐energy injuries, the ACL disrupts in the midsubstance [18].

A previous study that analysed the relationship between BB patterns and ACL tear location did not find an association between BB in the lateral compartment and ACL tear location [41]. These results may be influenced by the inclusion of patients with open physes. Skeletally immature patients show different BB patterns with a lower BB prevalence, which may be due to the shock‐absorbing properties of the physis [26]. The same study showed that BB involving the medial and lateral compartments was related to midsubstance ACL tears [41]. However, these results could not be reproduced in the current study, which did not show an association between the presence of BB in the MFC or MTP and ACL tear location. Various mechanisms for the development of medial‐sided BB have been discussed. Kaplan et al. suggested a contrecoup mechanism after a pivot‐shift injury with a compensatory varus alignment and medial rotation [12]. In contrast, other studies have suggested that an injury mechanism involving less valgus rotation and higher load in the sagittal plane may be the cause of BB in the medial compartment [42, 45]. The results of a video analysis study also showed that axial compression immediately after initial ground contact is more likely to be responsible for the BB in the medial compartment [9]. Furthermore, additive tibial internal rotation reduces the pressure on the MTP compared to an isolated compression force [22]. In summary, the results of the BB analysis suggest that the ACL tear location may depend on the biomechanical injury mechanism. Lower energy or a lower knee valgus angle could be possible causes for a more proximal ACL tear. In addition, this study provides diagnostic clues in MRI reading of BB distribution and ACL tear location, which may aid decision‐making in the preoperative selection of treatment strategy in daily clinical practice.

The distribution of ACL tear types in this study is consistent with previous studies, indicating that type III tears are the most common. However, a higher incidence of type I and type II tears (16% and 27% vs. 7% and 17%, respectively) has been reported [20]. The preclinical selection process for ACL‐preserving surgery could be a reason for the higher incidence of proximal tears [18]. Another possible explanation may be that the present study is the first to describe the distribution of ACL tear types using the measurement technique of Vermeijden et al. [40]. The median ACL tear location of 68.2% indicates that numerous ACL tears are on the border between Type II and Type III tears. Subjective assessment of ACL tear type without measurement of the proximal and distal remnants may explain a different classification and should therefore be avoided due to the availability of a reproducible measurement technique of the ACL tear location. The lower incidence of type IV and type V tears in the present study may be explained by the exclusion of young patients with open growth plates. Yet, these patients have a higher incidence of distal ACL tears [19].

This study has several limitations. First, selection bias cannot be excluded due to the retrospective nature of the study. The absence of severe injuries such as multi‐ligamentous knee injuries and the large number of skiing injuries are the result of patient‐recruiting process from a secondary care facility. Second, the use of MRI scans from different institutions with various sequences may have influenced the BB depth analysis. To account for potential variability in MRI, a semi‐quantitative method was used to analyse the BB depth. Third, MRI‐based categorization of ACL tear location was not confirmed intraoperatively due to the retrospective study design. Finally, the limitations of individual research approaches to analyse injury mechanisms have been described [15]. Although BB are considered a footprint of the injury mechanism [35], they can only provide a static representation with limited information about the exact biomechanical loading pattern of the knee joint.

## CONCLUSION

ACL tear location after an acute non‐contact injury is associated with distinct patterns of BB distribution, particularly involving the lateral compartment, indicating that different injury mechanisms may lead to different ACL tear locations.

## AUTHOR CONTRIBUTIONS

All authors contributed to the conception and design of the study. Materials preparation and data collection were performed by Steffen T. Ubl and Romed P. Vieider. Measurements were performed by Hannes Platzgummer, Christian Gaebler and Steffen T. Ubl. The first draft of the manuscript was written by Steffen T. Ubl. Romed P. Vieider, Jesse Seilern und Aspang, Hannes Platzgummer and Christian Gaebler made critical revisions to the structure of the article. Steffen T. Ubl and Jesse Seilern und Aspang guided the statistical methods and data processing. All authors commented on earlier versions of the manuscript. All authors read and approved the manuscript.

## CONFLICT OF INTEREST STATEMENT

The authors declare no conflict of interest.

## ETHICS STATEMENT

Approval was obtained from the institutional review board Medical University of Vienna. Informed consent is not applicable.

## Supporting information

Supporting information.

## Data Availability

The data sets utilized during the current study are available from the first author (ublsteffen@gmail.com) upon reasonable request.
