# Supplementary material for: Bone bruise distribution predicts anterior cruciate ligament tear location in non‐contact injuries
Source: J Exp Orthop. 2024 May 13;11(3):e12034. doi: 10.1002/jeo2.12034 (PMC11089843; doi:10.1002/jeo2.12034)
Supplement: Supplementary file 1 — Supporting information. [file JEO2-11-e12034-s001.docx]

Bone Bruise Distribution predicts Anterior Cruciate Ligament Tear Location in Non-Contact Injuries

Supplementary file 1: Prevalence and depth of bone bruises in the four anatomical sites and the segments. n (%)

|  | Bone bruise Prevalence | Bone bruise Depth | | | | |  |
| --- | --- | --- | --- | --- | --- | --- | --- |
|  |  | Superficial | Shallow | Deep | Extensive | Generalized | |
| MFC | 73 (46.2) | 4 (5.5) | 30 (41.1) | 29 (39.7) | 8 (11.0) | 2 (2.7) | |
| anterior | 1 (1.4) | 0 | 0 | 1 (100) | 0 | 0 | |
| central | 60 (82.2) | 4 (6.7) | 28 (46.7) | 22 (36.7) | 6 (10.0) | 0 | |
| posterior | 13 (17.8) | 0 | 2 (15.4) | 6 (46.2) | 3 (23.1) | 2 (15.4) | |
| MTP | 82 (51.9) | 0 | 9 (11.0) | 14 (17.1) | 24 (29.3) | 35 (42.7) | |
| anterior | 8 (9.8) | 0 | 0 | 3 (37.5) | 4 (50.0) | 1 (12.5) | |
| central | 5 (6.1) | 0 | 4 (80.0) | 0 | 0 | 1 (20.0) | |
| posterior | 76 (92.7) | 0 | 5 (6.6) | 14 (18.4) | 23 (30.3) | 34 (44.7) | |
| LFC | 100 (63.3) | 4 (4.0) | 13 (13.0) | 35 (35.0) | 26 (26.0) | 22 (22.0) | |
| anterior | 0 | 0 | 0 | 0 | 0 | 0 | |
| central | 99 (99.0) | 4 (4.0) | 13 (13.1) | 35 (35.4) | 25 (25.3) | 22 (22.2) | |
| posterior | 2 (2.0) | 0 | 1 (50.0) | 0 | 1 (50.0) | 0 | |
| LTP | 149 (94.3) | 0 | 1 (0.7) | 3 (2.0) | 24 (16.1) | 121 (81.2) | |
| anterior | 3 (2.0) | 0 | 0 | 2 (66.7) | 0 | 1 (33.3) | |
| central | 1 (0.7) | 0 | 1 (100) | 0 | 0 | 0 | |
| posterior | 149 (100) | 0 | 1 (0.7) | 3 (2.0) | 24 (16.1) | 121 (81.2) | |
| LFC, Lateral femoral condyle; LTP, Lateral tibial plateau; MFC, Medial femoral condyle; MTP, Medial tibial plateau | | | | | | | |
